# An examination of the temporal and geographical patterns of psychiatric emergency service use by multiple visit patients as a means for their early detection

**DOI:** 10.1186/1471-244X-7-60

**Published:** 2007-10-29

**Authors:** Yves JA Chaput, Marie-Josée Lebel

**Affiliations:** 1 Department of Psychiatry, Douglas Hospital, McGill University, Montreal, Quebec, Canada; 2 (At the time of this study) Notre-Dame Hospital, University of Montreal, Montreal, Quebec, Canada; 3 (At the time of this study) Department of Psychiatry, Notre-Dame Hospital, Montreal, Quebec, Canada

## Abstract

**Background:**

Frequent users of the psychiatric emergency service (PES) place a heavy burden upon the mental health care delivery system. The aim of this study was to identify distinct temporal or geographical patterns of PES use by these patients as potential markers for their early detection.

**Methods:**

Diagnostic profiles were obtained for patients making an intermediate (4 to 10) or a high (11 or more) number of visits to a general hospital PES in Montreal (Canada) between 1985 and 2004. Between-group comparisons were made with regards to several parameters. These included the time intervals between consecutive visits, visit clustering (single, repeating, and the time interval to the first cluster) and visits made to three other services where data was similarly acquired from 2002 to 2004.

**Results:**

The two multiple visit groups differed with regards to diagnostic profiles and actual time between consecutive visits (significantly shorter in patients with 11 or more visits). Patients with 11 or more visits were more likely to have a single cluster (3 or more visits/3 months) or repeating clusters (4 visits/3 months) in their patterns of use. Personality disorders were more prevalent in patients with single clusters as they were, along with schizophrenia, in those with repeating clusters. In addition, clusters were found to occur sufficiently early so as to be potentially useful as markers for early detection. Ten percent of those with 11 or more visits and 16% of those with an intermediate number of visits frequented at least one other PES. A small number of patients, primarily those with substance abuse, made over 50% of their visits to other services.

**Conclusion:**

Temporal and geographical patterns of use differed significantly between the multiple visit groups. These patterns, combined with distinct diagnostic profiles, could potentially lead to the more rapid identification and treatment of specific sub-groups of multiple visit patients.

## Background

A relatively small number of patients account for a disproportionate number of psychiatric emergency room visits and thus represent a substantial burden upon this service [1-7]. The psychiatric emergency service (PES) is an important gateway into the mental health system and the early detection of these patients could be of clinical and administrative importance to overall mental health care delivery. Several studies have shown that heavy and intermediate PES users possess distinct diagnostic profiles [5-7]. These profiles become more heavily weighted with chronic and severe psychopathology as the number of visits increases [6]. Although distinct, these profiles are by themselves not sufficiently unique to be used as potential markers for early multiple visit patient detection. Correlating them with distinct patterns of PES use, such as temporal or geographical patterns, may provide useful additional information.

With regards temporal patterns of use the majority of return visits to the PES occur within 3 months of the first, a finding that appears independent of whether patients ultimately make a high or an intermediate number of return visits [6,8]. Pasic et al. [7], found that using various definitions of what constitutes a high frequency user (partly on the basis of temporal patterns of use) resulted in different patient profiles. Differences however were mainly observed with regards variables such as enrollment in a mental health plan, homelessness and history of incarceration, variables that might not possess the same degree of pertinence from one country to another or, from one metal health care delivery system to another. As for geographical variables Oyewumi et al. [4] reported that up to 23% of their multiple visit patients (in Saskatoon, Canada) visited several, rather than the same PES. Whether this subgroup of multiple visit patients differed diagnostically from those making all of their visits at the same PES was not assessed.

There were several aims to the present study. First, to assess whether the diagnostic profiles of different multiple visit patient groups could be matched to distinct temporal or geographical patterns of PES use. If so, could this additional information be of use in their early detection.

## Methods

### Data collection

As previously described [6,9], clinical and demographic data were obtained from all adult patients visiting the PES of a university teaching hospital in Montreal (Canada) from June 15, 1985, to June 15, 2004. The database originated June 15 1985 as an 'in-house' register of seven variables (name, sex, service sector, referral source, disposition, date and time of entry into and, departure from, the PES) kept by the nursing staff. It was gradually expanded to include 63 additional clinical (including diagnostic) and demographic variables by July 1, 1996. At this time it was used for research purposes until December 31, 2000. After this date only the nursing register data was collected up until September 2002. The full database was used once again (until June 15, 2004) when this site, as well as three other PESs, participated in assessing the multiple PES patterns of use. Overall, 19,740 patients made 38,420 visits (2 ± 3.5 visits per patient) during the 19-year period. Multiple visit patients were divided into an intermediate group (4 to 10 visits, 1408 patients, 8025 visits, 5.7 visits per patient) and, a heavy user group (11 or more visits, 373 patients, 7789 visits, 21 visits per patient), in accordance with previous reports [6,7]. The average number of visits of the intermediate group approximated twice that of the standard deviation for all patients during the collection period and was equivalent to the 'high utilizers by standard deviation' group described by Pasic et al. [7]. Those with 1 to 3 visits (17,959 patients, 22,606 visits, 1.3 visits per patient) were included as a reference group. Data entry for the research database was performed by designated members of the nursing staff and by the principal investigator.

The 70 variables in the research database above were listed in a paper format and used as a triage instrument, for a two-year period beginning September 2002, for all patients visiting three other services. All services had strict rules regarding their respective geographical cathment areas that included procedures for integrating patients that did not have a fixed address (see [10] for a detailed discussion on sectors). The completed forms were forwarded to the principal investigator for data entry. The mains site, as well as two of the additional sites, were located in general hospitals and by protocol patients were triaged in the medical emergency department by both the nursing and medical staff and, if warranted, a psychiatric evaluation was requested. The fourth site was within a traditional psychiatric institute and operated as a walk-in clinic.

Diagnostic profiles were produced by attributing a "most probable" primary diagnosis, which was the diagnosis most frequently given during a patient's multiple visits. As over 60% of PES visits have been shown to occur during the daytime hours [6] only services that were covered by experienced, regular daytime psychiatric staff were included in this study. This was done in order to minimize diagnostic uncertainty. To further reduce diagnostic uncertainty diagnoses were grouped into broad categories. The latter differ from our previous report [6] as bipolar disorders and paranoid psychoses are now separate from the 'affective disorders' and 'schizophrenia' categories, respectively. Lastly, multiple-visit patients became increasing familiar to the regular PES staff, who routinely referred them to multidisciplinary treating teams (psychiatrists, social workers, nurse clinicians, psychologists) responsible for both their outpatient and inpatient care. These teams were primarily based upon the notion of maintaining continuity of care. As previously reported, over 80% of heavy users at the main site were, at one point during the time they frequented this PES, under the care of such teams [6]. During the multi center phase of this study 77% of heavy users and 67% of intermediate users were, at least at one point in time, under multidisciplinary outpatient care. As such, any further diagnostic uncertainty could be clarified by discussions with the treating team.

### Primary data analysis

Data were analyzed by using the statistical analysis program Systat (Version 11). Categorical variables (such as the overall diagnostic profiles) were analyzed using the "crosstabs" section for one- and two-way tables. Independence of cell frequencies was assessed by the Pearson chi square statistic. Odds ratios (OR), adjusted for sex and age using the logistic regression modules, were used to assess the association between a given diagnosis (independent variable) and either frequency of PES use (dependent variable) or cluster frequency (independent variable). The associated 95% confidence intervals of all ORs were assessed and did intersect 1.0 (only P values are presented). ORs were not adjusted for race as Caucasians represented over 90% of the sample. Analyses of variance were used in order to assess whether the means of numerical variables differed significantly. Simple linear regression (Least Squares method with resulting squared multiple R values) was used to test the hypothesis that a significant relation exists between numerical variables.

This study was approved by the institutional review board (IRB) scientific subcommittees at all sites and exempted from full review other than at one site, where full IRB approval was required.

## Results

Significant differences between the diagnostic profiles of the two multiple visit groups and, between the intermediate group and patients making less than 4 visits, are shown in Table 1. Each column represents a broad diagnostic category. Cells that are formatted in bold, italic and underlined represent significant differences between the formatted cell and the cell immediately underneath.

**Table 1:** Diagnostic profiles^ab ^of the different patient groups where a primary diagnosis could be ascertained^c^.

**Group**	**Adj**	**Pd**	**Dep**	**Bipd**	**Anx**	**Sa**	**Sch**	**Pnos**	**Omd**	**ParP**	**Other**	**Patients**
1 to 3 visits	***17%***	***10%***	14%	***11%***	***7%***	22%	***9%***	5%	2%	2%	2%	100% = 7765
4 to 10 visits	***4%***	15%	***7%***	16%	***4%***	***20%***	***28%***	***3%***	1%	2%	0.5%	100% = 1046
11+ visits	0.3%	17%	2%	19%	2%	14%	43%	0.6%	2%	0.3%	0%	100% = 343
Patients	15%	10%	13%	12%	6%	21%	13%	5%	2%	2%	0.9%	100% = 9154
(Visits)	(11%)	(14%)	(10%)	(12%)	(5%)	(20%)	(18%)	(4%)	(2%)	(2%)	(1%)	

^a ^Adj   Adjustment disorders, OR 0.06 (P < 0.001, group '1 to 3' vs group '4 to 10'), OR 0.24 (P < 0.001, '4 to 10' vs group '11+').

Pd      Personality disorders, OR 3.7 (P < 0.001, group '1 to 3' vs group '4 to 10').

Dep    Depression, ORs 0.7 (P < 0.05, group '4 to 10' vs group '11+').

Bipd   Bipolar disorders, ORs 0.77 (P < 0.01, group '1 to 3' vs group '11+').

Anx    Anxiety disorders, ORs 0.61 (P < 0.001, group '1 to 3' vs group '4 to 10'), OR 0.43 (P < 0.01, group   '4 to 10' vs group '11 +').

Sa      Substance abuse, ORs 0.65 (P < 0.005, group '4 to 10' vs group '11 +').

Pnos  Psychosis not otherwise specified, OR 7.6 (P < 0.005, group '4 to 10' vs group '11 +').

Sch    Schizophrenia, OR 3.6 (P < 0.001, group '1 to 3' vs group '4 to 10'), OR 2.0 (P < 0.001, group '4 to 10' vs group '11+').

^b ^ORs were not calculated for columns with patient totals 2% or less (Omd = organic mental disorders, ParP = paranoid psychoses, Other = all other diagnoses). A cell is formatted in '**bold**/underlined/*italic*' if the OR of having a given diagnosis is significantly different from that of the cell immediately underneath.

^c ^Ascertained for 7765 of 17,959 patients with 1 to 3 visits, 1046 of the 1408 patients with 4 to 10 visits and, 343 of the 373 patients with 11+ visits. The sum of the percentages for each row may not equal 100% due to rounding.

To determine whether the multiple visit groups differed with regards the time intervals between consecutive visits the first nine intervals (up to the tenth visit) were each coded as falling within a given number of months of the preceding visit. Time to the second or, time between the second and third visit did not differentiate between the multiple visit groups. Thereafter however the intervals were significantly shorter in the heavy PES user patients (see Table 2 for an example). Second, the actual time (in days) of each time interval was assessed and significant differences between the two multiple visit groups for all visit intervals emerged (Table 3).

**Table 2:** The time interval (in months) between the 3^rd ^and 4^th ^visits for the two multiple visit groups^a^.

	1 month	3 months	6 months	12 months	18 months	24 months	30 months	36 months	48 months	> 48 months	N (patients)
4 to 10	25 %	16 %	13 %	15 %	8 %	5 %	4 %	3 %	4 %	7 %	1406^b^
11+	33 %	20 %	14 %	17 %	7 %	3 %	1 %	2 %	1 %	2 %	373
N	**477**	**306**	**241**	**269**	**132**	**80**	**55**	**43**	**58**	**118**	**1779**

^a ^Pearson Chi-square value (46.3), df (17), P < 0.001. Percentages = % of patients within each user group with intervals falling within the number of months in the column heading.

^b ^Date and time of entry to the PES could not be obtained for 2 of the 1408 patients making 4 to 10 visits.

**Table 3:** Average^a ^(± SE) time (in days) between subsequent visits for patients in the multiple visit groups.

	**1^st ^to 2^nd^**	**2^nd ^to 3^rd^**	**3^rd ^to 4^th^**	**4^th ^to 5^th^**	**5^th ^to 6^th^**	**6^th ^to 7^th^**	**7^th ^to 8^th^**	**8^th ^to 9^th^**	**9^th ^to 10^th^**
1 to3	605 ± 15	593 ± 25							
	(N = 3642)^b^	(N = 1001)							
4 to 10	446 ± 25	397 ± 21	433 ± 17	405 ± 20	342 ± 20	353 ± 22	315 ± 31	410 ± 41	443 ± 54
	(N = 1406)	(N = 1406)	(N = 1406)	(N = 904)	(N = 622)	(N = 424)	(N = 258)	(N = 139)	(N = 54)
11+	335 ± 37	274 ± 32	199 ± 34	212 ± 30	187 ± 25	183 ± 24	196 ± 26	170 ± 25	186 ± 20
	(N = 373)	(N = 373)	(N = 373)	(N = 373)	(N = 373)	(N = 373)	(N = 373)	(N = 373)	(N = 373)

^a ^The mean values for all adjacent cells within a column differ significantly (ANOVA, P < 0.001 for all except the following three adjacent cells. That between the '4 to 10' and the '11 +' group at the 1^st ^to 2^nd ^(P < 0.01), the 2^nd ^to 3^rd ^(P < 0.005) and at the 7^th ^to 8^th ^visits (P < 0.005).

^b ^N values in parentheses represent the number of patients.

Shorter time intervals between visits in patients with higher visit counts suggest that these patients might also differ with regards visit clustering. A single cluster was defined as 3 visits within a 3-month period (type 1 cluster) or, 3 visits within a 6-month period (type 2 cluster). Only 38% (N = 536) and 55% (N = 769) of the intermediate users versus 82% (N = 306) and 94% (N = 352) of those in the heavy user group had at least one type 1 or one type 2 cluster during their many visits, respectively. Larger cluster sizes of 6 (type 1a) or 7 (type 1b) visits per 3-months were also examined. This required isolating a cohort of 258 patients with 8 or more visits from the intermediate group as an adequate comparison group to the 373 patients with heavy PES use. Odds ratios showed that those in the heavy use group (independent variable) were six and fourteen times more likely to have a type 1a or a type 1b cluster (dependent variable), respectively (P < 0.005).

We examined the possibility that the higher number of patients with a cluster in the heavy use group might primarily reflect the overall greater number of visits made by these patients. In this case, the presence of a cluster, cluster size and cluster frequency should be related to the visit count. Linear regression of the number of patients with a type 1 cluster and the visit count for patients with 5 or more visits (N = 1277) showed a very strong relationship (P < 0.005, squared multiple R value of 0.84). Interestingly, regressing cluster sizes ranging from 0 (none) to 7 visits per 3-months (dependent variable) and visit count for patients with 8 or more visits (N = 631) showed a very weak relationship (squared multiple R value of 0.2). The frequency with which a 4 visits per 3-months cluster repeated itself in patients making 8 or more visits also showed a significant relationship to the visit count (P < 0.005, squared multiple R value of 0.7). As expected, the average number of visits per patient (21 visits/patient) for those with repeating clusters (N = 289 patients) was much higher than for those without (12 visits/patient, N = 342 patients). Repeating clusters of 7 visits per 6-months were also examined in 247 patients with 14 or more visits extracted from the heavy use group. Again, cluster frequency showed a significant (P < 0.005, squared multiple R value of 0.7) relationship with visit count and those (N = 89) with repeating clusters had a greater average number of visits per patient than those without (35 versus 20 visits/patient, respectively).

The relationship between clustering (a single or repeating clusters) and the diagnostic profile was examined. The presence of at least one cluster of three or more visits per 3-months was examined in all patients with 4 or more visits from both multiple visit groups (N = 1389). Those with a cluster (N = 661, of which 63% consisted of one single cluster) had a slight (19% with versus 12% without), albeit significant increase in personality disorders (OR of 1.6, P < 0.005) than those without a cluster (N = 728). Table 4 illustrates the diagnostic profiles of patients with and those without repeating clusters. The group with the highest number of repeating clusters (cohort b) includes almost exclusively personality disorders and patients with schizophrenia. No relationship was found between the size of a single cluster and the diagnostic profile.

**Table 4:** Relationship between cluster frequency and diagnosis^a ^for two cohorts^b ^of selected multiple visit patients.

	**Personality disorders**		**Bipolar disorders**		**Subs. abuse disorders**		**Schizophrenia**		**Other disorders**		**Patients^c ^(visits)**	
Cohort	a	b	a	b	a	b	a	b	a	b	a	b
No clusters	31	21^d^	71	30	40	14	131	75	24	9	297	149
	(341)	(453)	(840)	(619)	(458)	(262)	(1687)	(1519)	(250)	(152)	(3576)	(3005)
1 to 3 clusters	50	17	31	5	38	12	63	24	24	4	206	62
	(875)	(473)	(535)	(111)	(499)	(281)	(1101)	(772)	(303)	(79)	(3313)	(1716)
≥ 4 clusters	14	9	3	2	7	1	23	8	1	0	48	20
	(771)	(603)	(179)	(149)	(180)	(29)	(1106)	(500)	(26)	0	(2262)	(1281)
Total patients	95	47	105	37	85	27	217	107	49	13	551	231
(Total visits)	(1987)	(1529)	(1554)	(879)	(1137)	(572)	(3894)	(2791)	(579)	(231)	(9151)	(6002)

^a ^Other = adjustment disorders, depression, anxiety disorders, organic mental disorders, psychosis not otherwise specified, paranoid psychoses.

^b ^Cohort a = repeating clusters (4 visits in 3 months) in patients with 8 or more visits taken from the intermediate and heavy user groups. Cohort b = repeating clusters (7 visits in 6 months) in patients with 14 or more visits taken from the heavy user group.

^c ^N values = 551 (of the 631 cohort a patients) and 231 (of the 247 cohort b patients) are patients that had a clear diagnosis.

^d ^The numbers above the parentheses represent the patient count, the numbers in the parenthesis represent the corresponding number of visits.

That some diagnoses are more prevalent in patients who exhibit a clustering pattern of PES use would be more clinically pertinent if the cluster occurred sufficiently early within the total time span over which a patient visited the PES. We therefore assessed the time at which the first cluster occurred (of any size or frequency, starting with a minimum type 1 cluster) in relation to the total time span for completing all visits for a selected group of multiple visit patients. Three groups were chosen. Patients with 4 visits (N = 766, 118 of which had clusters), 10 visits (N = 73, 37 of which had clusters) and those with 40 or more visits (N = 29, all had clusters). The average time span for each patient group to complete all of their visits was 720, 2504 and 5125 days, respectively whereas the average time to complete the first cluster was 357, 1050 and 654 days, respectively.

We next examined whether the patterns of PES use by multiple visit patients included admissions at other services. Data was acquired at services that were 11 km, 39 km and 256 km away from the main site. A subgroup of multiple visit patients at the main site was isolated using the criteria that their last visit fell within the time period of data acquisition for the multi center trial (N = 268 for the intermediate group and N = 132 for the heavy use group). Overall, 16% (N = 42) of the intermediate group patients and 10% (N = 13) of those with heavy PES use frequented at least one other service (between group differences were not significant).

We next examined whether some patients primarily visit multiple, rather than a single PES. Of the 12,083 diagnosed patients frequenting all 4 services during the two-year multi center phase of this study173 had 50% or more of their visits at the other services. Locally (either of the four services) their count was low (2 ± 3 visits per patient) but it increased to 4 ± 3 visits per patient when the non-local visits were included. Their clinical profile differed from that of either of the two multiple visit groups or, that of the 11,910 patients visiting a single one of the four sites. There was a predominance of substance abuse patients (30% versus 16% for the single site patients, OR of 2.3, P < 0.005) and patients with schizophrenia (20% versus 13% for the single site patients, OR of 1.7, P < 0.05).

## Discussion

Using a prolonged observation period as well as an extensive division of psychiatric diagnoses this study adds further detail to the previously described relationship between visit frequency and diagnostic profile in the PES [1,6,7]. Patients with adjustment, anxiety and depressive disorders and patients with psychoses of unknown etiology primarily made a low (1 to 3) number of visits. Personality and substance abuse disorders spanned both the low and intermediate range of PES use whereas patients with schizophrenia were primarily intermediate and heavy PES users. This latter finding was observed despite the fact that paranoid psychoses were excluded from the schizophrenia category, slightly reducing its proportion in comparison to our previous report [6]. Although diagnostic certainty in the PES has been questioned and remains a potential weakness of this as well as of all PES studies, schizophrenia, compared to other psychiatric diagnoses, has been shown to possess the greatest stability in different clinical settings [11,12]. The present study also highlights the relative contribution of bipolar disorders to intermediate and heavy PES use (Table 1), a finding in keeping with their extensive use of inpatient and outpatient services [13,14].

Temporal patterns of PES use also differed between the multiple visit groups. Patients in the heavy PES user group had significantly shorter time intervals between their consecutive visits. They were also more likely to exhibit a single 'cluster' pattern of service use than patients in the intermediate group. This finding could, at least in part, be attributable to the fact that heavy users made (by definition) a much higher number of visits. The only diagnostic category more predominant in patients with primarily single clusters (compared to those without clusters) was personality disorders. This was so even in the intermediate PES user group. Cluster frequency, although not cluster size, also correlated with visit frequency. However, repeating clusters of even small sizes (4 visits per 3 months) require a high number of visits (8 or more) and by and large these patients would have already been tagged heavy PES users. Nevertheless, this parameter does appear to provide additional clinical information inasmuch as the diagnostic profile of heavy users with a high level of clustering differed from the overall heavy user group profile. As opposed to the general profile, heavy users that cluster frequently were almost equally likely to have a personality disorder than to suffer from schizophrenia. They also made a higher average number of visits per patient than heavy users without repeating clusters. Some of these patients may be detectable at the intermediate PES user level as suggested by the higher proportion of personality disorders in patients with predominantly single clusters. In addition, clusters can be observed at an early stage, long before a patient's total number of visits have been completed.

Geographical patterns of PES use also appeared to provide potentially useful clinical information. Noteworthy was the substantial number of visits made by patients in both the intermediate and heavy PES user groups to multiple services. Oyewumi et al. ([15]) reported that 23% of frequent users made visits to multiple PESs whereas the figure reported here was closer to 14%. However, the three participating services in this study were chosen because they were either structurally or functionally dissimilar to the main site, not because they were the closest. Several other services were located in the main site's vicinity. As such it is more than likely that the percents reported here for both multiple visit groups frequenting other services are underestimations.

In addition, a subgroup of multiple visit patients was characterized by having 50% or more of their visits at other services. This subgroup appeared to possess a diagnostic profile different from that of the other multiple visit groups. This particular diagnostic profile was marked by a preponderance of substance abuse disorders. In the present study patients with substance abuse were not typically found to be heavy users of a single PES. Combining all user groups (including patients with single visits) they nevertheless represented one of the largest patient categories. Their total number of visits equaled that made by patients with schizophrenia, the heaviest of PES users (Table 1). Patients with substance abuse have also been shown to contribute significantly to the frequent user phenomenon in the medical emergency department [16]. As such, the overall burden to both emergency services represented by these patients would appear to be considerable. That they tend to visit multiple services suggests that this broad diagnostic category might be substantially underestimated if assessed purely at the local level.

## Conclusion

Patients making a very high number of PES visits are much easier to describe that to treat. Many in the present study were (or had been) under active out/inpatient multi disciplinary care. An underlying premise of this study is that long-term data acquisition can lead to a more precise clinical description of the various PES user groups (and subgroups). This could ultimately lead to the development of treatment strategies tailored towards the common elements within these groups, rather than strategies geared towards each individual patient.

Clinically, the probability of finding a cluster (and ultimately, repeating clusters) at the local level rises along with the visit count. This relationship parallels that between 'visit count and diagnostic profile', at least until extremely high levels of PES use are reached. Making use of both findings, targeting specific diagnostic groups (personality disorders, schizophrenia) when clusters are present in their patterns of use could potentially help detect future heavy PES users at a much earlier stage. Early detection could help in the acquisition of large volumes of data for the study of group characteristics. On the policy side the above findings illustrate the pertinence of planning for long-term data acquisition in the PES, both locally and regionally, as it is highly unlikely that such levels of detection could be achieved using a 'paper' format. The finding that some frequent user subgroups visit several services further underscores this need. Better regional integration of individual PESs and, a more efficient means of communication with certain community services, especially those servicing substance abusers, might be considered. Alternatively, consolidating several services to a smaller number might offer substantial advantages in terms of quality of care and costs containment.

## List of abbreviations

PES = Psychiatric Emergency Service

OR = Odds Ratio

IRB = Institutional Review Board

## Competing interests

The author(s) declare that they have no competing interests.

## Authors' contributions

As primary author for this article, YC takes full responsibility for the design of the trial, for data acquisition, data analysis and data interpretation and the writing of the manuscript.

M-JL, the chief coordinator of the trail, had significant input as to the design of the database and had full responsibility for the actual implementation of the study at all sites. She also had significant input as to the interpretation of the results and the conceptualization (not the writing) of the manuscript.

## Pre-publication history

The pre-publication history for this paper can be accessed here:


